# Supplementary material for: Molecular characterization and prospective isolation of human fetal cochlear hair cell progenitors
Source: Nat Commun. 2018 Oct 2;9:4027. doi: 10.1038/s41467-018-06334-7 (PMC6168603; doi:10.1038/s41467-018-06334-7)
Supplement: Supplementary file 1 — Supplementary Information [file 41467_2018_6334_MOESM1_ESM.pdf]

# Molecular characterization and prospective isolation of human fetal cochlear hair cell progenitors

**Roccio et al.**

**Supplementary figure 1: In Situ Hybridization (RNAscope) for human LGR5**

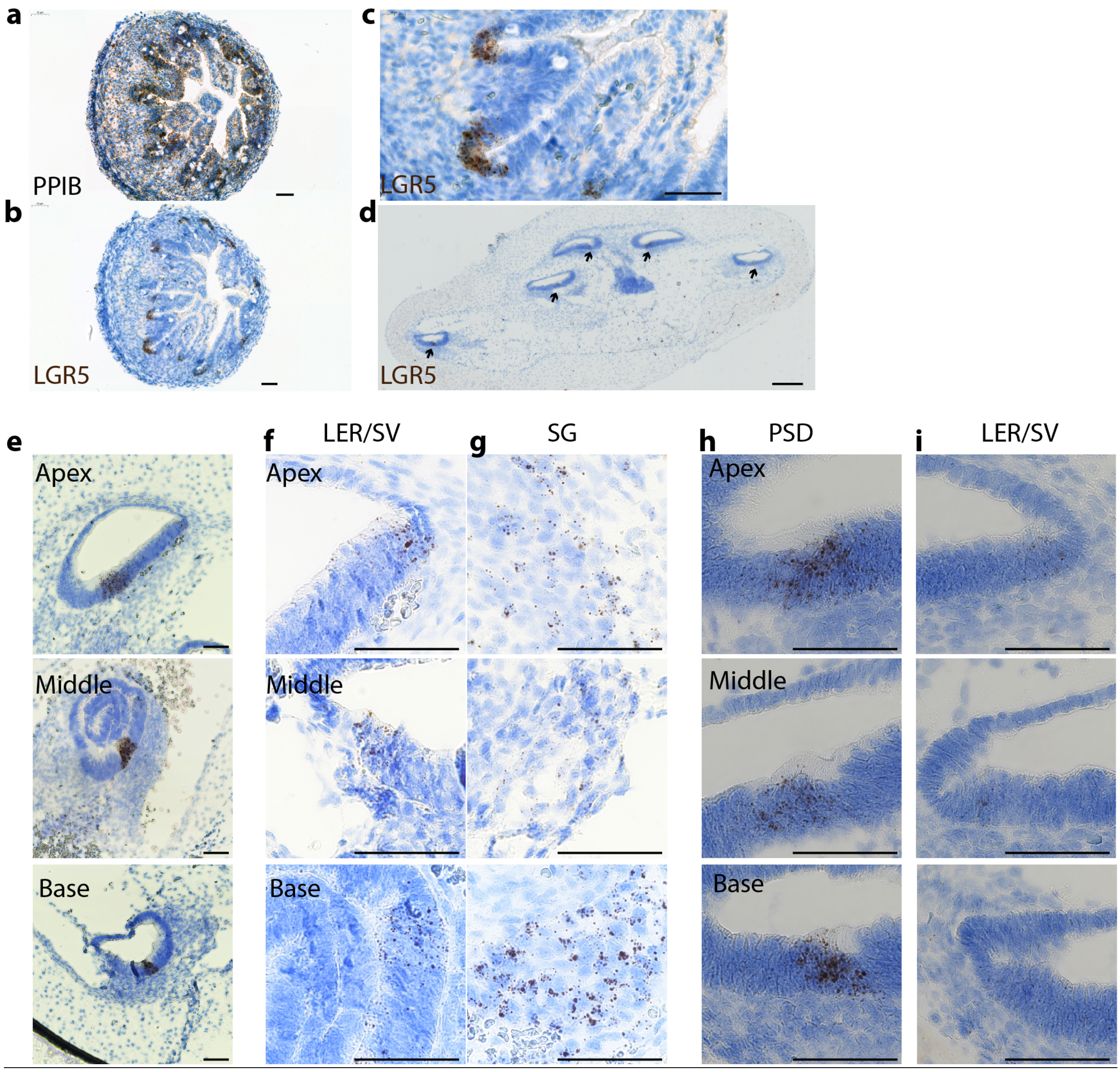


**Supplementary figure 1**

(**a-c**) Human fetal intestine at W9.5-10 of development (E1273). In Situ Hybridization (ISH) for *PPIB* (housekeeping gene) and *LGR5* is shown as brown dots. Haematoxylin staining (in blue) to illustrate tissue morphology. Scale bar= 50µm c) Higher magnification of the intestinal crypts showing positivity for *LGR5*. Scale bar= 50µm**.**
**(d)** Fetal cochlea at W9.3 of development (E1278). ISH positive signal for *LGR5*, indicated by arrows. Scale bar= 200µm
(**e-g**) *LGR5* ISH in a human cochlea W9.2 (E1276)**.** (**e**) Shown are apical, middle and basal turns. (**f**) Higher magnification of the cochlear duct in the region of the lesser epithelial ridge/spiral ligament/stria vascularis (LER/SV). Spiral ganglion (SG) is shown in (**g**).
(**h-i**) *LGR5* ISH in a human cochlea W9.3 (E1278)**.** Apical, middle and basal turns are shown. PSD (h) and lesser epithelial ridge/spiral ligament/stria vascularis (LER/SV) (i) are shown. Scale bar= 50µm

**Supplementary Figure 2 : Hair cell staining vestibular sensory epithelia**

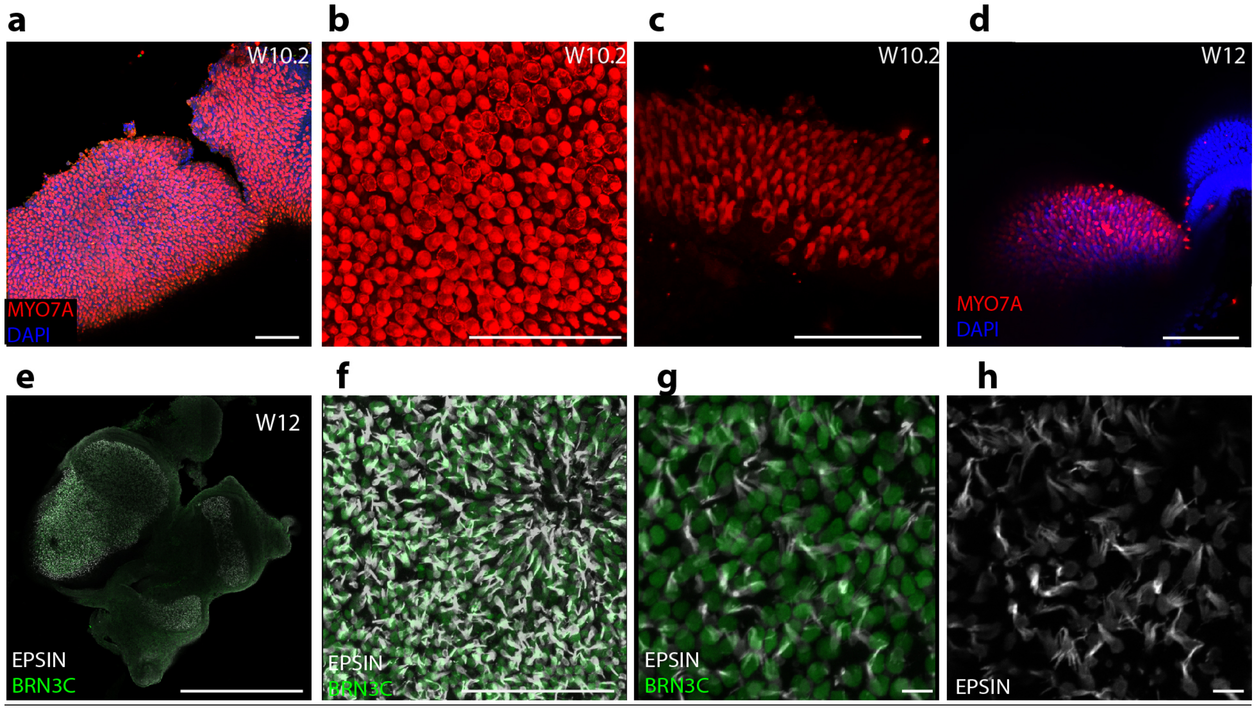


**Supplementary figure 2**

(**a**) Whole mount confocal imaging of a human utricle at week 10.2 of development (sample E1213). The sample is immunostained for MYO7A (red). Nuclei are labeled by DAPI (blue). Scale bar =100 μm.

(**b**) Higher magnification of the same sample. Scale bar =100 μm.

(**c**) Whole mount confocal imaging of a human ampulla at week 10.2 of development immunostained for MYO7A (red). Nuclei are labeled by DAPI (blue). Scale bar =100 μm.

(**d**) Whole mount confocal imaging of a human ampulla at week 12.1 of development immunostained for MYO7A (red). Nuclei are labeled by DAPI (blue). (sample E1210). Scale bar =100 μm.

(**e**) Whole mount confocal imaging of a human utricle and ampullae at week 12 of development (sample E1289) immunostained for BRN3C (green) and ESPIN (white). Scale bar =1mm.

**f-h**) Details of the utricle sensory epithelium. Scale bar =100 μm (f), Scale bar =10 μm (g-h)

## Supplementary figure 3: Surface marker characterization

**a**

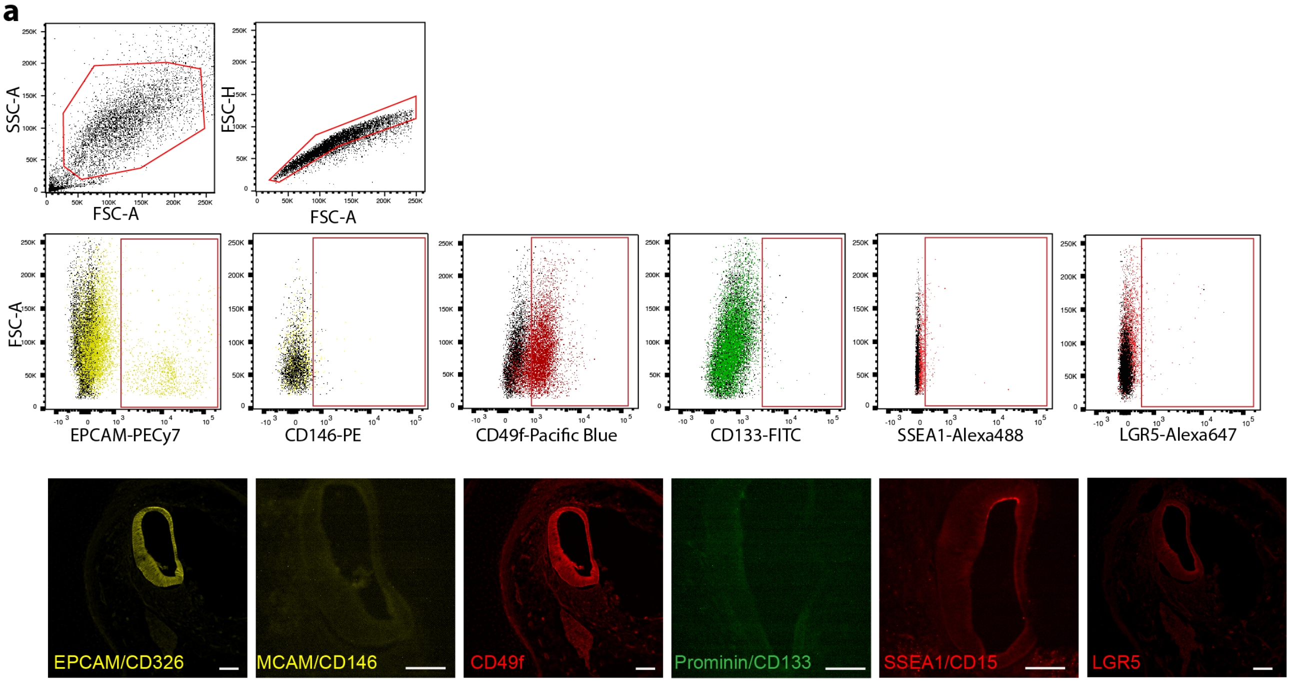


**b**

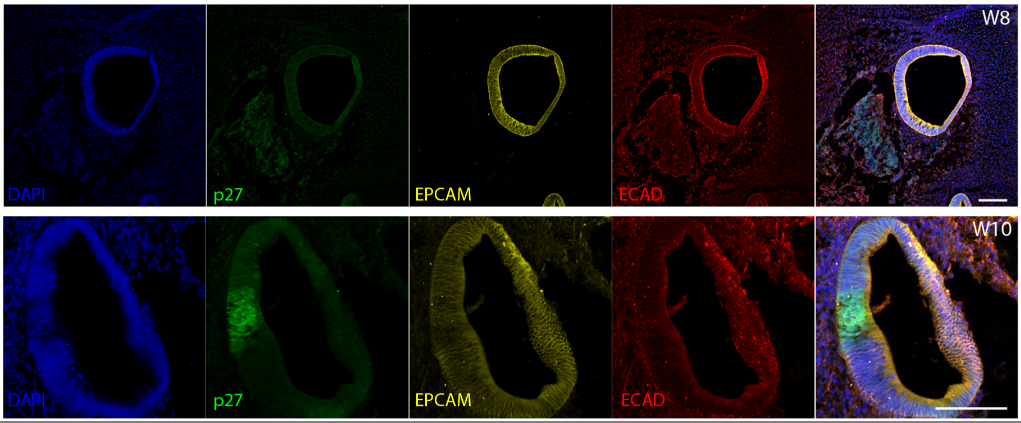


**Supplementary figure 3**

(**a**) Flow cytometry analysis for the markers EPCAM/CD326, MCAM/CD146, CD49f, Prominin/CD133, SSEA1/CD15 and LGR5. Samples (E1213, E1206 and E1299). Representative example of the gating strategy is shown in upper panel for gates p1(left) and p2(right). Lower panels show cells from p2 gates with unstained samples (black) and stained populations (in color) for each marker. Representative images of basal turn cochlear duct immunostained for EPCAM/CD326, MCAM/CD146, CD49f, Prominin/CD133, SSEA1/CD15 and LGR5 (sample E1201) are shown. Scale bar =100 μm.

(**b**) EPCAM, ECAD and p27Kip1 co-staining of cochlear duct samples at W8 (E1202) and W10 (E1201) of development. Scale bar =100 μm.

**Supplementary figure 4: Spiral ganglion characterization**

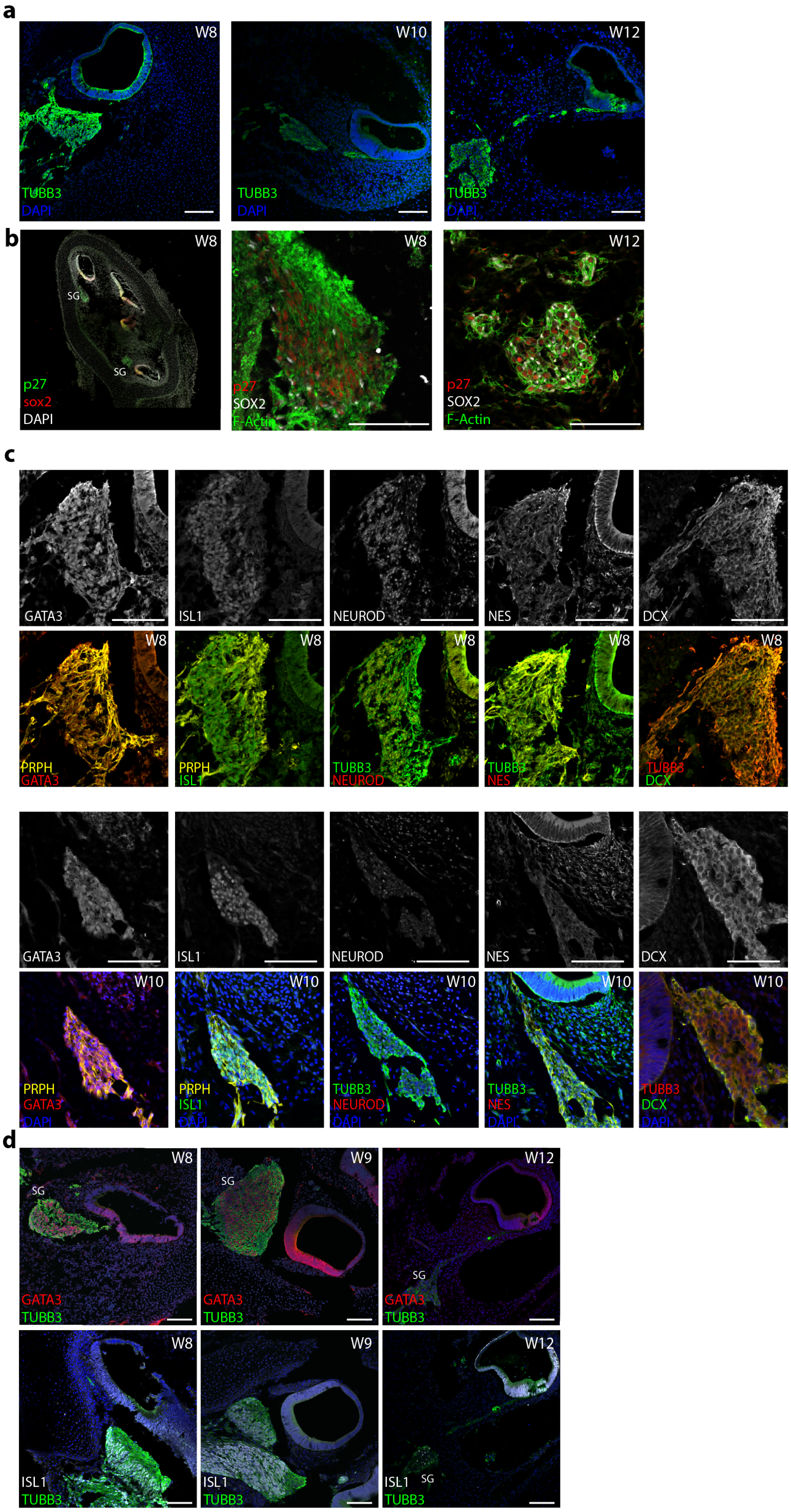

**Supplementary figure 4**

(**a**) Representative images of 3 stages of development (W8, W10 and W12). Cochleas were cyosectioned and immunostained for the neuronal marker βIII Tubulin (TUBB3) (green). The basal turn is shown. Scale bar =100 μm.

(**b**) Immunostaining of W8.4 (sample E1251) cochlea for SOX2 (red) and p27Kip1 (green) shows the lack of SOX2 expression in the p27+ SG cells. Higher magnification of the ganglion at W8.4 (E1251) and W12 (E1203) are shown. Scale bar =100 μm.

(**c**) Cochlear tissue immunostained at W8 (E1202) and W10 (E1201) for GATA3, Islet1 (ISL1), NEUROD, Nestin (NES) and Doublecortin (DCX). βIII tubulin (TUBB3) or peripherin (PRPH) staining is used to visualize the ganglion. Scale bar=100μm

(**d**) Immunostaining of W8 (E1202), W9 (E1228) and W12 (E1203) cochlear tissue for GATA3, TUBB3 or Islet1 and TUBB3. The cochlear duct also expresses these two markers at all time points analyzed. Scale bar =100 μm.

**Supplementary figure 5: Organoids culture validation using Lgr5-GFP murine supporting cells.**

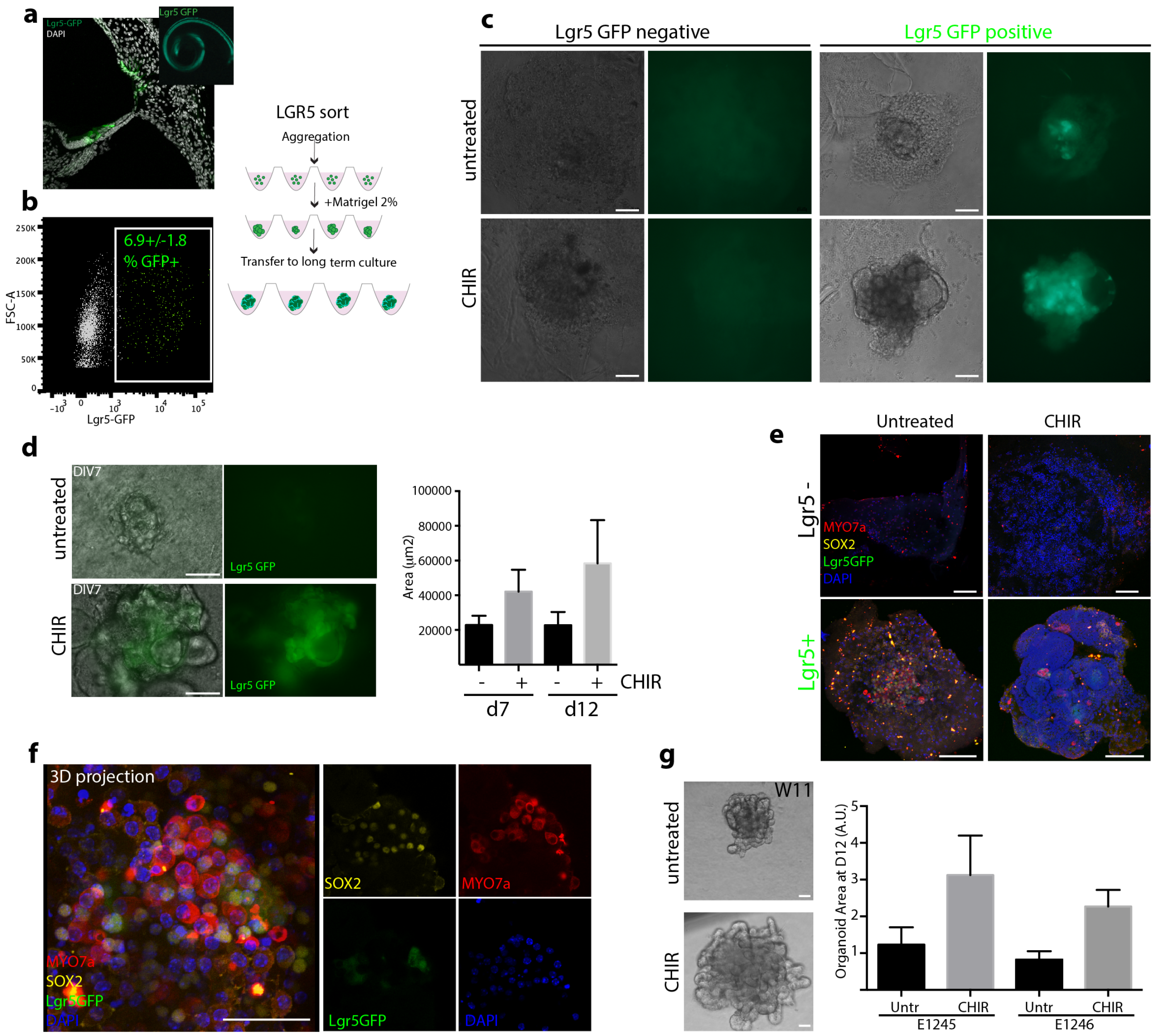


**Supplementary figure 5**

(**a**) GFP expression in Lgr5-GFP reporter mice labels supporting cells in the organ of Corti, cross section and a whole mount preparation are shown.

(**b**) Flow cytometry analysis and FACS sorting of the GFP positive cells isolated from the early postnatal murine sensory epithelium and schematic of the re-aggregation protocol and organoid generation.

(**c**) Epithelial organoids expressing GFP can be expanded by CHIR99021 (CHIR) supplementation. Lgr5-GFP negative cells, grow largely as mesenchyme in these culture conditions. Scale bar=100µm.

(**d**) Assessment of the organoids area at day 7 and day 12 in culture in presence/absence of CHIR99021. Bar graph shows mean+/- s.d. N=4 organoids per condition/time point are assessed. Scale bar = 100µm.

(**e**) Hair cell differentiation is assessed at day 15-20 *in vitro* by immunostaining the organoids for Myo7a (red) and sox2 (yellow). Hair cell-like cells are obtained only from the GFP+ organoids (n=3 independent experiments). Scale bar=100µm

(**f**) Representative example of hair cells derived from the Lgr5-GFP+ sorted cells. 3D projection (left) and a selected single stack for the separate channels are shown (right). Scale bar=50µm.

(**g**) Assessment of organoid size for two independent W11 human fetal samples (E1245 and E1246) in presence/absence of CHIR99021 added at 3µM twice during two weeks expansion. 8 organoids per sample per treatment were assessed. Bar graph shows Mean+/- s.d. Scale bar=100µm.

**Supplementary figure 6: EPCAM negative cell differentiation**

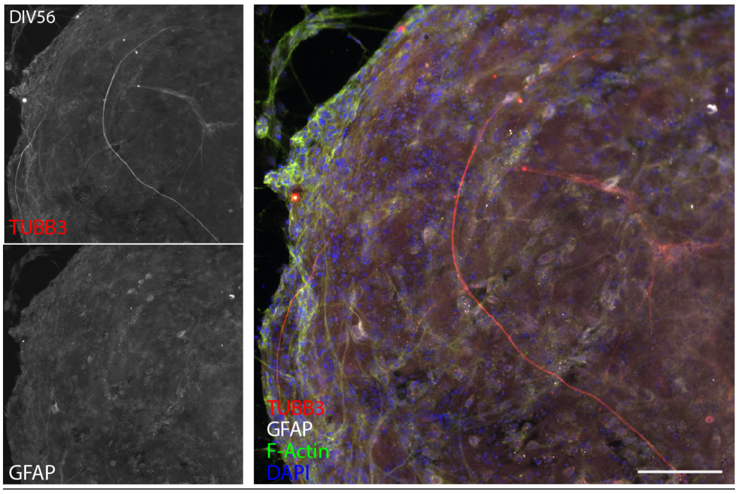


**Supplementary figure 6**
Immunostaining for βIII Tubulin (TUBB3) (red) and GFAP (green) in organoids derived from sorted EPCAM- cells, cultured for 56 days *in vitro* (sample E1220). Scale bar =100 μm.

**Supplementary figure 7: Sorting strategy to isolate PSD resident cells**

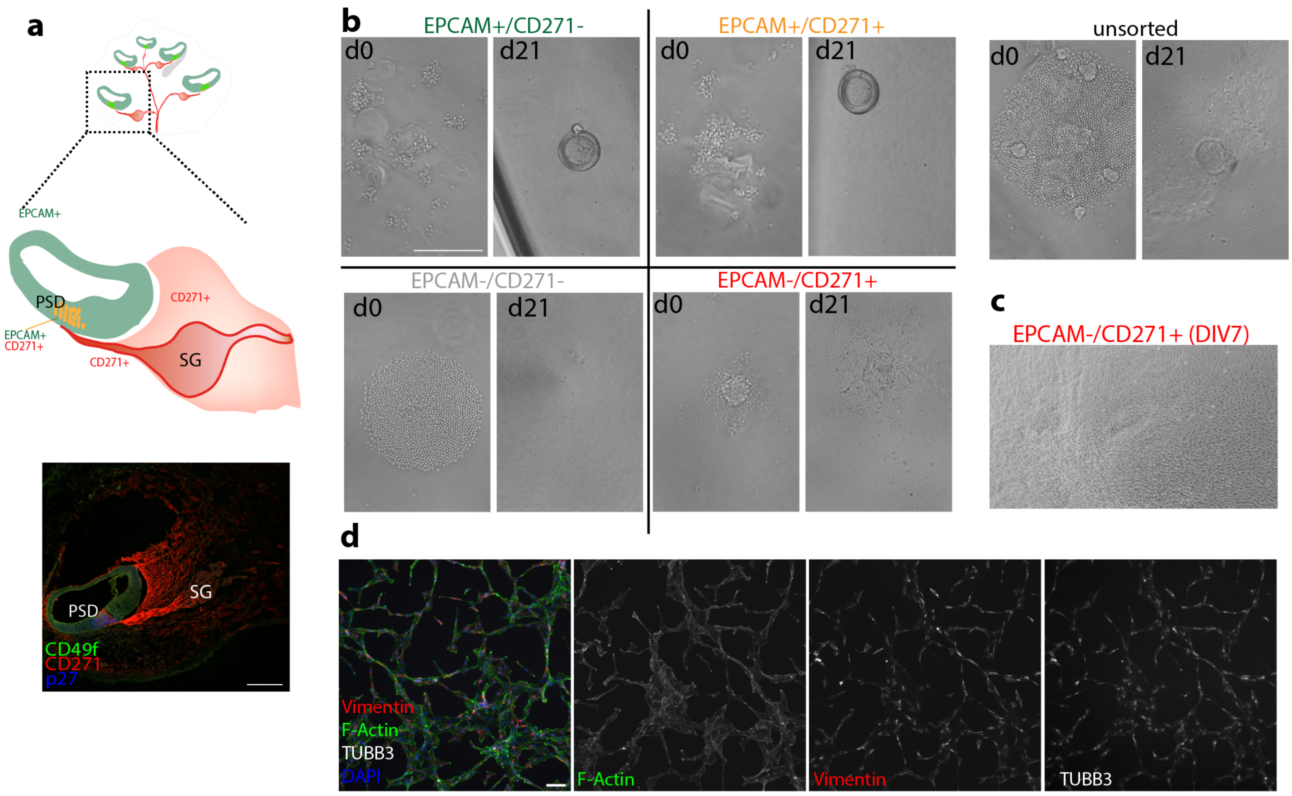


**Supplementary figure 7**

(**a**) Schematic representing the expression pattern of the selected markers EPCAM and CD271 and immunostaining of a W10 cochlea sample. The PSD is visualized by SOX2 staining (blue). CD271 expression is not restricted to the SG, but also expressed by mesenchymal tissue in the region of the developing spiral limbus starting from W10 of development. Scale bar =100 $\mu$m

(**b**) Representative images of the cultures derived from the four sorted populations immediately after sort (day 0) and at day 20 *in vitro*. The unsorted cell pool undergoing the same procedure is shown for comparison. (sample E1219). Scale bar =100 $\mu$m

(**c**) Representative image of a confluent culture derived from EPCAM-/CD271+ cells after 1 week in culture.

(**d**) Representative images of EPCAM-/CD271+ derived cultures immunostained for Vimentin (red), $\beta$III Tubulin (TUBB3) (gray) and co-labelled with phalloidin and DAPI. Scale bar =100 $\mu$m.

**Supplementary figure 8: Characterization EPCAM+/CD271+ organoids**

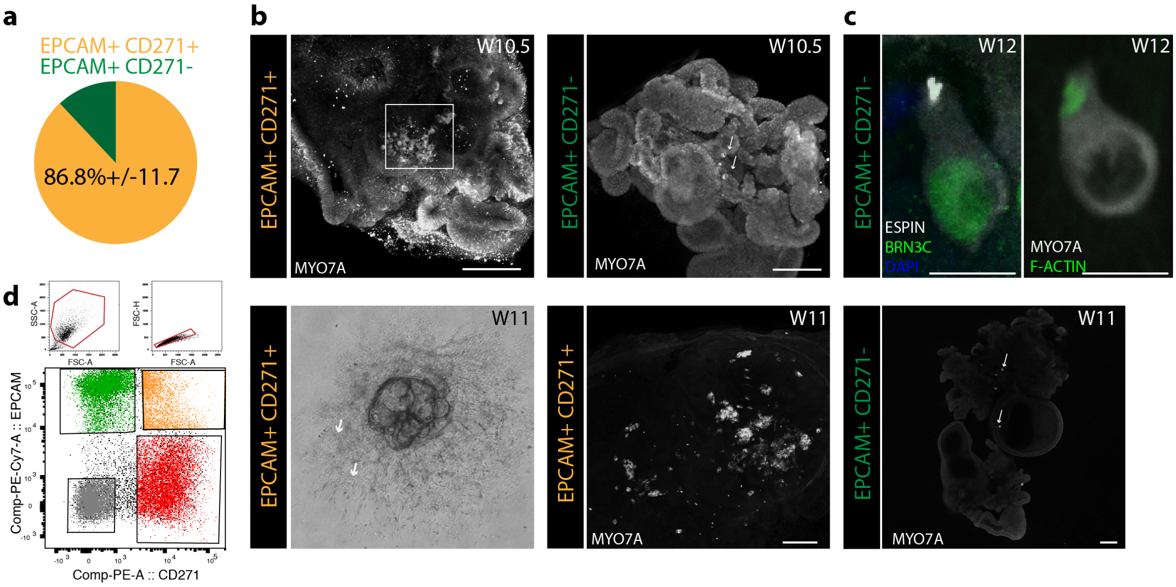


**Supplementary figure 8**

(**a**) Quantification of the contribution to total hair cell-like cell generation from the two sorted populations. N=5 different fetal sample. Values are mean +/- s.d.

(**b**) Immunostaining of EPCAM+/CD271+ (left) and EPCAM+/CD271- (right) organoids for MYO7A (sample E1270). Scale bar=100µm. Arrows point at single MYO7A+ cells obtained in the single positive population.

(**c**) Representative images of ESPIN and BRN3C immunostaining and MYO7A and F-Actin staining in cells derived from EPCAM+/CD271- organoids (sample W12, E1289) Scale bar=10µm.

(**d**) Example of FACS sorting based on EPCAM and CD271 co-staining (W11 sample E1254). Gating strategy indicated on top. Yellow gate (double positive) including cells expressing lower level of EPCAM leads to isolation cells which eventually outgrow with a mesenchymal phenotype as shown in the light microscopy image and indicated by the arrows. Right panels show immunostaining for MYO7A in organoids derived from EPCAM+/CD271+ or EPCAM+/CD271- sorted cells (W11 sample E1254). Maximum intensity projection of the confocal stack is shown. Scale bar=100µm.

**Supplementary figure 9: Functional characterization of the in vitro generated hair cell-like cells**

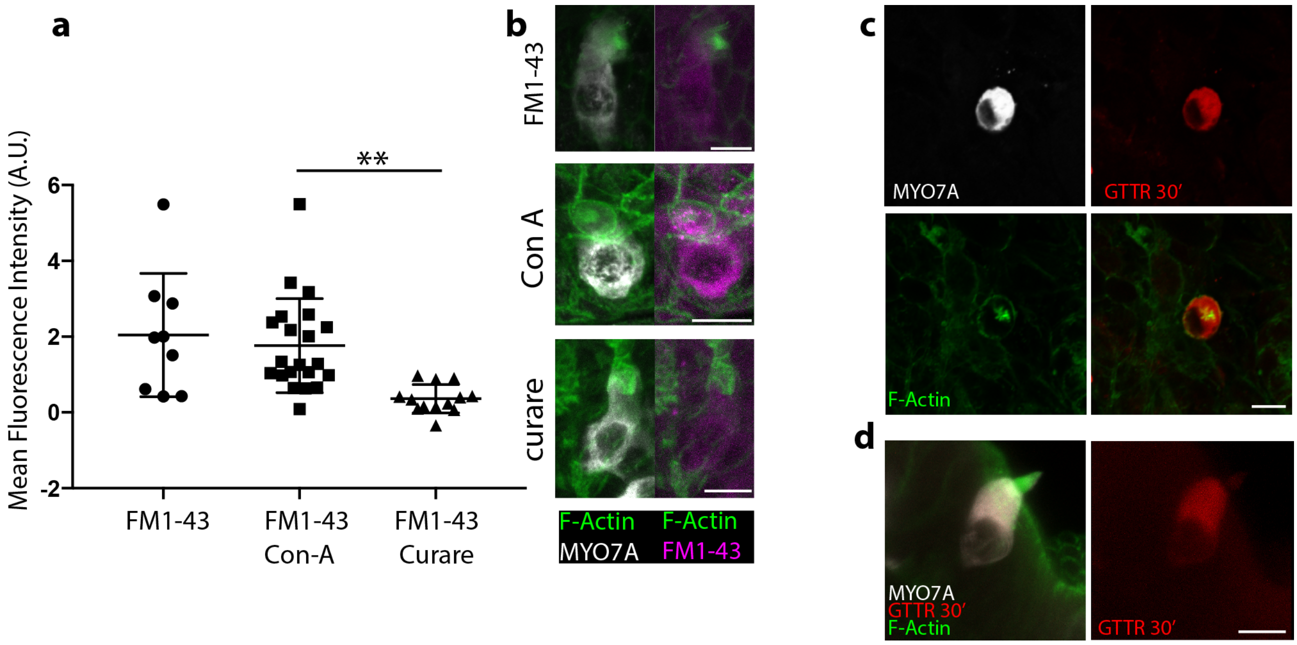


**Supplementary figure9**
(**a**) Quantification of FM1-43 loading in hair cell-like cells derived from a W12 sample (E1289).
Cells were pretreated with concanavalin A or curare for 10 minutes or left untreated prior to the application of FM1-43 for 30 seconds. Fluorescence intensity (mean gray value) for the FM1-43 channel was quantified in the volume identified by MYO7A staining using FIJI and background corrected.
Samples were prepared in parallel and imaged by confocal scanning in sequential mode using the same parameters. FM1-43-only: n=9 cells from 2 different organoids, Curare-FM1-43: n=13 cells from 2 different organoids; Concanavalin A-FM1-43: n=21 cells from 3 different organoids. Unpaired t test (** $p<0.01$)
(b) Representative images of the hair cell-like cells quantified in the 3 conditions. Merged channels for F-Actin and MYO7A and F-Actin and FM1-43 are shown. Scale bar=10μm.
(**c-d**) GTTR loading in hair cell-like cells from a W9.6 sample (E1286). Single channels and merged image are shown for two different morphologically distinct hair cells types. Scale bar=10μm.

## SUPPLEMENTARY TABLES

### Supplementary Table 1: Human fetal samples used in the study

| sample ID | Week P.C. | experiment | |
|---|---|---|---|
| Histochemistry | | | |
| E1209 | 7.7 | immunostaining/Cryosection | |
| E1202 | 7.9 | immunostaining/Cryosection | |
| E1251 | 8.4 | immunostaining/Cryosection | |
| E1241 | 9.1 | immunostaining/Cryosection | |
| E1228 | 9 | immunostaining/Cryosection | |
| E1192 | 10.3 | immunostaining/Cryosection | |
| E1201 | 10.3 | immunostaining/Cryosection | |
| E1213 | 10.2 | immunostaining/Cryosection | |
| E1195 | 10.9 | immunostaining/Cryosection | |
| E1210 | 12.1 | immunostaining/Cryosection | |
| E1203 | 12 | immunostaining/Cryosection | |
| E1274 | 6 | immunostaining/Cryosection | ** |
| E1257 | 11.6 | immunostaining/Cryosection | ** |
| In Situ Hybridization (ISH) | | | |
| E1273 | 9.5-10* | intestine ISH | |
| E1276 | 9.2 | cochlea ISH Lgr5 | |
| E1278 | 9.3 | cochlea ISH Lgr5 | |
| EPCAM+ sort organoids | | | |
| E1253 | 10.4 | differentiation to hair cells with co-culture | |
| E1248 | 11.4 | differentiation to hair cells with co-culture | |
| E1246 | 11.5 | differentiation to hair cells with co-culture | |
| E1245 | 11.4 | differentiation to hair cells with co-culture | |
| E1229 | 11 | differentiation to hair cells with co-culture | |
| E1224 | 8.9 | short term culture or diff with other protocols | |
| E1220 | 9.3 | short term culture or diff with other protocols | |
| E1237 | 9.7 | short term culture or diff with other protocols | |
| E1232 | 9.7 | short term culture or diff with other protocols | |
| EPCAM+/CD271+ sort organoids | | | |
| E1254 | 11 | differentiation to hair cells with co-culture | |
| E1242 | 12.5 | differentiation to hair cells with co-culture | |
| E1238 | 9.7 | differentiation to hair cells with co-culture | |
| E1270 | 10.5 | differentiation to hair cells with co-culture | |
| E1273 | 9.5-10* | differentiation to hair cells with co-culture | |
| E1286 | 9.6 | differentiation to hair cells with co-culture | |
| E1289 | 12 | differentiation to hair cells with co-culture | |
| E1227 | 10.4 | flow cytometry only | |
| E1225 | 9.9 | flow cytometry only | |
| E1221 | 9.8 | flow cytometry only | |
| E1214 | 8.5 | flow cytometry only | |
| E1219 | 9.4 | flow cytometry only | |
| Gene expression Fluidigm | | | |
| E1236 | 9 | | |
| E1235 | 11.8 | | |
| E1208 | 8.3 | | |
| E1204 | 11.1 | | |
| Flow cytometry | | | |
| E1206 | 11.1 | Flow cytometry | |
| E1213 | 10.2 | Flow cytometry | |
| E1199 | 10.5 | Flow cytometry | |

notes

* calculated 9.1, p.m. W12, morphology W10

** only for staining controls

**Supplementary Table 2: antibodies used in the study**

| antigen | | conjugated | cat# | company | dilution /final conc. |
|---|---|---|---|---|---|
| **CD49f/α6Integrin** | Rat IgG2a, κ | eFluor 450 | 48-0495 | eBioscience | 1 : 100 |
| **CD271/p75** | Mouse IgG1, κ | Alexa fluo 647 | 560877 | BD | 1 : 100 |
| **CD271/p75** | Mouse IgG1k | PE | 557196 | BD Pharmigen | 1 : 100 |
| **Lgr5** | Rat IgG2b, λ | alexa Fluor 647 | 562912 | BD | 1 : 100 |
| **CD146/MCAM** | Mouse IgG1k | PE | 561013 | BD | 1 : 100 |
| **CD326/EPCAM** | Mouse IgG1, κ | PE-Cy7 | 25-9326 | eBioscience | 1 : 100 |
| **CD15/SSEA1** | Mouse IgM | Alexa 488 | 60060AD.1 | stem cell technologies | 1 : 100 |
| **CD133/Prominin** | mouse IgG1κ | VioBright FITC | 130-105-225 | Milteny | 1 : 100 |
| **p27** | Rabbit monoclonal | | ab32034 | AbCAM | 1 : 200 |
| **sox2** | Rabbit polyclonal | | 48-1400 | Invitrogen | 1 : 100 |
| **sox2** | Mouse IgG1k | | 561469 | BD | 1 : 100 |
| **sox2** | Mouse IgG2b | | MAB4343 | Millipore | 1 : 200 |
| **Ki67** | Rabbit polyclonal | | NCL-ki67p | Novocastra | 1 : 100 |
| **Myo7a** | Rabbit polyclonal | | 25-6790 | Proteus | 1 : 200 |
| **Jag1** | Rabbit polyclonal | | AB7771 | ABCAM | 1 : 100 |
| **Jag1** | mouse IgG1k (E12) | | SC-390177 | Santa Cruz | 1 : 100 |
| **βcatenin** | mouse | | 610154 | BD Transduction laboratories | 1 : 100 |
| **E-cadherin** | Rat IgG1 | | ab11512 | abcam | 1 : 200 |
| **FBXO2** | Rabbit monoclonal | | ab133717 | AbCAM | 1 : 100 |
| **FBXO2** | mouse (A-12) | | sc-393873 | Santa Cruz | 1 : 100 |
| **FBXO2** | goat (D-19) | | sc-69400 | Santa Cruz | 1 : 100 |
| **ZO-1** | Rabbit policlonal | | AB59720 | ABCAM | 1 : 100 |
| **Sox9** | Rabbit monoclonal | | ab185966 | AbCAM | 1 : 100 |
| **Nestin** | Mouse IgG1, κ | | 611658 | BD Transduction Laboratories | 1 : 200 |
| **Peripherin** | Rabbit polyclonal | | AB1530 | Millipore | 1 : 100 |
| **GATA3** | Mouse monoclonal IgG1 | | MA1-028 | Thermofisher scientific | 1 : 100 |
| **Islet1** | Mouse IgG2b | | 39.4D5(sup) | DSHB | 1 : 10 |
| **NeuroD** | Rabbit polyclonal | | AB16508 | ABCAM | 1 : 100 |
| **Doublecortin** | Goat | | SC8066 | Santa Cruz | 1 : 100 |
| **βIII tubulin** | Mouse IgG2a | | MAB1195 | R&D | 1 : 200 |
| **Vimentin** | Mouse IgG1k | | SC6260 | Santa Cruz | 1 : 100 |
| **Espin** | Rabbit polyclonal | | | Gift Jim Bartles | 2.5mg/ml |
| **BRN3C** | Mouse monoclonal IgG1 | | SC81980 | Santa Cruz | 1 : 100 |
| **Fluorescently labeled compounds** | | | | | |
| **FM1-43 FX** | | | F35355 | Thermofisher scientific | 5μM |
| **Gentamycin-Texas Red** | | | | Gift Anthony Ricci | 0.3mg/ml |
| **Phalloidin** | | ATTO-488 | 49409 | Sigma | 1 : 100 |
