# Supplementary material for: Molecular characterization and prospective isolation of human fetal cochlear hair cell progenitors
Source: Nat Commun. 2018 Oct 2;9:4027. doi: 10.1038/s41467-018-06334-7 (PMC6168603; doi:10.1038/s41467-018-06334-7)
Supplement: Supplementary file 3 — Description of Additional Supplementary Files [file 41467_2018_6334_MOESM3_ESM.pdf]

Description of Additional Supplementary Files

File Name: Supplementary Data 1
Description: Gene expression data from the 96.96 gene array.
Indicated are the mean Ct values obtained in the assay from 3 technical replicates, corrected for the expression levels of beta-actin (*ACTB*) and *GAPDH* (ΔCt). Columns are ranked with highly expressed genes on top.
Two cochlear duct (CD) samples, two utricle (UT) samples and two spiral ganglion (SG) samples from the same donors at week 8.3 (W8.3) and week 11.1 (W11.1) are analyzed. For the spiral ganglion, two additional samples are analyzed: SG_W9 and SG_W11.8

File Name: Supplementary Data 2
Description: List of primers used in the study for the 96.96 gene array.
Gene symbol, plate and forward (FP) and reverse (RP) primers are indicated.
